# Persistence of the Oral Probiotic *Streptococcus salivarius* M18 Is Dose Dependent and Megaplasmid Transfer Can Augment Their Bacteriocin Production and Adhesion Characteristics

**DOI:** 10.1371/journal.pone.0065991

**Published:** 2013-06-13

**Authors:** Jeremy P. Burton, Philip A. Wescombe, Jean M. Macklaim, Melissa H. C. Chai, Kyle MacDonald, John D. F. Hale, John Tagg, Gregor Reid, Gregory B. Gloor, Peter A. Cadieux

**Affiliations:** 1 Canadian Research and Development Centre for Probiotics, Lawson Health Research Institute, London, Ontario, Canada; 2 Division of Urology, Department of Surgery, Western University, London, Ontario, Canada; 3 Department of Microbiology and Immunology, Western University, London, Ontario, Canada; 4 Department of Biochemistry, Schulich School of Medicine and Dentistry, Western University, London, Ontario, Canada; 5 Department of Microbiology and Immunology, University of Otago, Dunedin, New Zealand; 6 BLIS Technologies Ltd, Centre for Innovation, University of Otago, Dunedin, New Zealand; Teagasc Food Research Centre, Ireland

## Abstract

Bacteriocin-producing probiotic *Streptococcus salivarius* M18 offers beneficial modulatory capabilities within the oral microbiome, apparently through potent inhibitory activity against potentially deleterious bacteria, such as *Streptococcus pyogenes*. The oral cavity persistence of *S. salivarius* M18 was investigated in 75 subjects receiving four different doses for 28 days. Sixty per cent of the subjects already had some inhibitor-producing *S. salivarius* in their saliva prior to probiotic intervention. Strain M18’s persistence was dependent upon the dose, but not the period of administration. Culture analysis indicated that in some individuals the introduced strain had almost entirely replaced the indigenous *S. salivarius*, though the total numbers of the species did not increase. Selected subjects showing either high or low probiotic persistence had their salivary populations profiled using Illumina sequencing of the V6 region of the 16S rRNA gene. Analysis indicated that while certain bacterial phenotypes were markedly modulated, the overall composition of the oral microbiome was not modified by the probiotic treatment. Megaplasmids encoding bacteriocins and adhesion factors were transferred *in vitro* to generate a transconjugant *S. salivarius* exhibiting enhanced antimicrobial production and binding capabilities to HEp-2 cells. Since no widespread perturbation of the existing indigenous microbiota was associated with oral instillation and given its antimicrobial activity against potentially pathogenic streptococci, it appears that application of probiotic strain M18 offers potential low impact alternative to classical antibiotic prophylaxis. For candidate probiotic strains having relatively poor antimicrobial or adhesive properties, unique derivatives displaying improved probiotic performance may be engineered *in vitro* by megaplasmid transfer.

## Introduction

In the past, properties such as the impact of dose on probiotic persistence and modulation of the microbiota have been less frequently studied, as the major focus of probiotic research has been on achieving efficacious outcomes, often with the largest cost-effective dosage regimen. Probiotics are often touted as being like small factories producing biologically active substances that benefit the host, but as most probiotics rapidly transit through the oral and digestive tracts following their ingestion, the likelihood of persistence at their principal target site is low. Previous studies have found that probiotic bacteria do not generally persist for more than a few hours or days in the intestinal tract [1] or oral cavity [2], though there are some exceptions reported for the oral cavity and vagina [3], [4].

Bacterial pathogens tend to have specific virulence traits that facilitate their attachment and subsequent invasion, particularly of oral and intestinal tissue, even in the presence of a protective layer of commensal bacteria, which themselves have adapted for attachment and survival, yet, seldom do the same commensal species become established in detectable numbers when administered in probiotic formulations. This has led to the suggestion that host factors influence the persistence of a microorganism newly introduced to an already established microbial ecosystem. Studies in which probiotic strains do not persist have largely failed to elucidate whether this is because of colonisation resistance, damage to the probiotic strains during their preparation, host specific attachment incompatibilities, dosage deficiencies or other mechanisms [5], [6]. Interestingly, some probiotic bacteria do not appear to perform consistently well in clinical studies and there appear to be often ill-defined factors relating to the manufacturing process that can influence the subsequent performance of a microorganism *in vitro* or *in vivo* [5], [6], [7]. In practice most probiotic strains have been propagated *in vitro* many times prior to being used in scaled commercial fermentations. During the course of serial passaging, bacteria can slowly accumulate random mutations within their chromosomes or jettison rarely translated DNA [8], [9]. ‘Muller’s rachet’ theory holds that asexual organisms (in this case bacteria kept in pure culture that are not transferring or receiving DNA from their surrounding community), display genetic drift resulting in a loss of functions as a consequence of the gradual genetic decline effected by random mutation [9], [10].

As a result of the oral microbiota being implicated in a variety of systemic conditions [11], attempts are being made to address these through treating the oral cavity. The proximal location of the oral cavity provides comparatively uncomplicated access for probiotics and for sampling to assess their impact. Studies have shown that the microbial composition of saliva is derived from a variety of oral ecosystems, such as the dorsum of the tongue [12]. *Streptococcus salivarius* has an innate capability of binding to and persisting on the tongue dorsum and some strains release into saliva copious quantities of bacteriocins that could provide a targeted way of removing deleterious bacteria [4], [13], [14]. *In vivo* bacteriocin production is often cited as the principal means by which health benefits are realised following the consumption of probiotic products [15], [16]. The passage of these organisms through the oral cavity and gut up-regulates bacteriocin production by members of the host’s microbiota [17]. In order to achieve sustained bacteriocin release, persistence of the probiotic bacteria is likely necessary [18].

To date, almost all of the bacteriocin-producing capability of *S. salivarius* appears attributable to genetic determinants localized on a megaplasmid (size 160 to 220 kbp). Naturally occurring transmission of these bacteriocin-encoding megaplasmids has been shown to occur both *in vitro* and *in vivo*. Their highly-flexible propensity for acquisition, expression and de-commissioning of a wide variety of bacteriocin loci may help account for the numerical prominence of *S. salivarius* in the oral cavity and be a mechanistic basis for *S. salivarius* having a major role in the maintenance of a balanced oral ecosystem. It has been suggested that *S. salivarius* megaplasmids may function as repositories for bacteriocin determinants acquired from a variety of oral species via transposition of IS elements [13]. Preliminary molecular analyses have indicated that these megaplasmids can also encode molecules aiding host cell adhesion, without induction of antibiotic resistance [19].

The goal of the present study was to investigate the effect of different dosage levels of *Streptococcus salivarius* M18 on the persistence of these bacteriocin-producing cells. Bacteriocin and adhesion determinants have previously been identified on certain *S. salivarius* megaplasmids and the influence of inter-strain transfer of these on host cell adhesion and antimicrobial activity was evaluated using *in vitro* models for the purpose of tailoring probiotic strains.

## Materials and Methods

### Subjects

The protocol and consent documents were reviewed and approved by the New Zealand Lower South Regional Ethics Committee. Seventy-five subjects (average age of 19 years) were recruited from a University class of approximately 200 students. All subjects were older than 18 years of age and gave informed written consent. The subjects were randomized and blinded to one of four identical looking dosage groups (taking lozenges containing 1×10^6^ (*n = *19), 1×10^7^ (*n = *20), 1×10^8^ (*n = *17) or 1×10^9^ (*n = *19) colony-forming units [CFU] of *Streptococcus salivarius* M18 per dose). The subjects used one lozenge per day for 28 days after tooth brushing. Samples (ca. 1 ml) of unstimulated saliva were obtained upon entry to the study and then each week at least 12 hours after lozenge treatment.

The saliva samples were serially diluted in phosphate buffered saline (PBS) and 50 µl was spiral plated onto various agar media including Columbia blood agar base, supplemented with 0.1% (w/v) calcium carbonate and 5% (v/v) human blood [Fort Richards Laboratories, Ltd., Auckland, NZ] (BACa). Streptomycin (100 µg/ml) was added to facilitate detection of the marked streptomycin resistant *S. salivarius* strain M18, and Mitis Salivarius agar (MS, [Fort Richards Laboratories, Ltd.]) was used for the general selection and enumeration of *S. salivarius*. Agar cultures were incubated for 18 hours in air supplemented with 5% CO_2_ at 37°C. Forty colonies having the characteristic *S. salivarius* phenotype on the MS plate from each subject were picked into a lawn culture of *Micrococcus luteus* I1 that had just been freshly inoculated on BACa. This indicator bacterium is known to be sensitive to a wide variety of streptococcal bacteriocin-like inhibitory substances (BLIS). *Streptococcus*-like colonies that grew on BACa streptomycin (i.e. presumptive *S. salivarius* M18) were picked into a freshly seeded BACa lawn culture of *Streptococcus mutans* OMZ 175, an indicator strain uniquely sensitive to BLIS activities of strain M18 (Burton unpublished). Total *S. salivarius* populations from MS plates were also tested for bacteriocin like inhibitory substances by deferred antagonism testing [20].

### *Streptococcus Salivarius* Strains used in the Study

Strain M18 is used as probiotic and produces multiple bacteriocins [19], and strain M18^−/−^ is a megaplasmid-negative and thereby bacteriocin-deficient variant of strain M18, Strain M18^K12p^ is a derivative of strain M18 that has been cured of its original plasmid but now contains megaplasmid DNA acquired from *S. salivarius* K12. Strain K12, the prototype *S. salivarius* probiotic, produces a variety of megaplasmid-encoded bacteriocins including the lantibiotics salivaricin A and salivaricin B [8], [21]. Strain K12^−/−^ is a megaplasmid-negative variant of strain K12. Strain K12^M18p^ is a derivative of the megaplasmid –negative strain K12^−/−^ now containing the strain M18 megaplasmid. Strain JIM8777 (genome sequenced) [22], ATCC 7073^T^, JH (produces multiple bacteriocins) [8], Min5 (produces multiple bacteriocins) [21], ToveR, ToveS [23], [24], A-23-4 (salivaricin A only producer), NR [8], [25] DB (non producer), 20P3 [21], [26].

### DNA Purification and Sequence Analysis

Total DNA was extracted from 500 µl of sample saliva pre-incubated for 10 min at 37°C with 50 µl of 8.8 mmol/l dithiothreitol using the PureLink™ genomic DNA kit (Invitrogen, Auckland, NZ) as per the manufacturer’s instructions for Gram-positive bacteria. DNA was eluted from the column in 100 µl of elution buffer. The primers L-V6 (59-CAACGCGARGAACCTTACC-39) and R-V6 (59-ACAACACGAGCTGACGAC-39) were chosen to amplify the V6 hyper variable region of the 16S rRNA gene [27]. The following PCR reaction conditions were used: 5 units Taq platinum: 1.7 mM MgCl, 210 mM dNTPs and 640 nM of each primer. A touchdown protocol was employed with: initial denaturation 94°C for 2 min; denaturation 94°C; annealing starting at 61°C and dropping with 1°C over 10 cycles with the remaining 15 cycles at 51°C; extension at 72°C; all for 45 seconds and a final elongation step for 2 min. A negative control including all ingredients but with water instead of DNA template, and a positive control with a lower limit of detectable DNA, were performed alongside all test reactions. PCR-products were used when the negative control was free of PCR product and the positive control amplified. A constant volume aliquot of each amplification product was run on a 1.5% (w/v) agarose gel to determine the approximate amount of product and sent for sequencing at The Next-Generation Sequencing Facility (Illumina) in The Centre for Applied Genomics at the Hospital for Sick Children, Toronto, Canada. Data analysis was performed as previously described [28]. 16S rRNA Operational Taxonomic Units seed sequences were deposited in NCBI Short Read Archive with the BioSample accession SAMN02055382.

### *In vitro* Intra-species Transfer of Megaplasmids

Using differences in sensitivity to antibiotics and bacteriocin of both megaplasmid donor and recipient strains, the transcongugants were generated and confirmed by ERIC-PCR [8]. In brief, the plasmid donor strain (M18 or K12) was grown on CNA-P agar (a medium that represses salivaricin A [SalA] and salivaricin B [SalB] production) [29] while the recipient strain (M18^−/−^ or K12^−/−^, plasmid-negative derivatives resistant to 500 µg/ml streptomycin or spectinomycin) in brain heart infusion (BHI) (Difco, MD) for 18 h at 42°C in a candle jar. Plasmid donor cells were collected on a sterile cotton swab and resuspended in 5 ml fresh BHI. One milliliter of the plasmid recipient strain was added to the mixture and then incubated in a candle jar for 18 h at 42°C (incubation above 37°C represses SalA and SalB production). Positive controls were included in all experiments and consisted only of donor cells or recipient cells in 5 ml BHI. Samples of the control and test mixtures were taken at 0, 2, 4 and 18 h using a cotton swab that was dipped into the BHI culture and then used to swab the surface of a BACa^+Str^ plate. The plate was incubated for 18 h at 37°C in 5% CO_2_ in air.

Subsequent selection for SalA- and spectinomycin-resistant colonies was carried out as follows; *Streptococcus salivarius* A23-4, a SalA producer (and therefore possesses the SalA immunity genes) was inoculated diametrically across a BACa plate and incubated for 18 h at 37°C in 5% CO_2_ in air. Visible bacterial growth was removed the next day using the edge of a microscope slide, following which the plate surface was exposed to chloroform vapors for 30 min to kill residual producer cells. The plate was left to air for 1 h leaving only the deposited bacteriocin. A cotton swab charged with growth from each BACa^+Str^ plate was then used to streak across the sterilized BACa plates and the plate incubated at 37°C in 5% CO_2_ in air for 18 h. Thirty SalA-resistant colonies in this region were picked off and stab-inoculated onto a BACa plate pre-seeded with an *Micrococcus luteus* I1 lawn, which is highly sensitive to antimicrobial activities. The lawn was seeded by charging a cotton swab with a I1 THB culture (18 h, 37°C, 5% CO_2_ in air) and creating a confluent lawn over the entire BACa plate. This was then incubated for 18 h at 37°C in 5% CO_2_ in air. Donor and recipient cells were stab- inoculated onto each plate as positive and negative controls. SalA-resistant colonies which produced a detectable inhibition zone against I1 were then struck onto a BACa^+Spec^ plate (100 µg/ml) and incubated for 18 h at 37°C in 5% CO_2_ in air. Colonies which grew on the BACa^+Spec^ plate were considered to be recipient cells that had successfully acquired SalA-encoding DNA from the plasmid-containing donor strain (i.e. they were putative plasmid recipients). These were also checked for their bacteriocin-producing abilities (as described).

### Simultaneous Antagonism Testing of Recipient Strains with Transferred Megaplasmids

The testing for inhibitory activity of *S. salivarius* strains (K12, K12^−/−^, M18, M18^−/−^, M18^K12p^, K12^M18p^) against putative periodontal pathogens was carried out using the simultaneous antagonism method. All strains, except *Porphymonas gingivalis* and *Porphymonas canoris* were sub-cultured on BACa (Columbia Blood Agar Base [Difco, BD] supplemented with 0.1% (w/v) calcium carbonate, 5% (v/v) human blood [NZ blood service]), when required. *P. gingivalis* and *P. canoris* were sub-cultured on BACaHV (BACa supplemented with 5 µg/ml hemin (Hemin chloride, bovine [Sigma]) and 1 µg/ml menadione (Vitamin K [Sigma]). Bacterial suspensions of *P. gingivalis* strains JK45, W50, *P. intermedia* strains ATCC 25611 and BGBL and *P. canoris* strains P21 in 3 ml THB (Todd Hewitt Broth [Bacto, BD]) were used to make a lawn on blood-based agar (*P. gingivalis* strains were assessed on both BACa and BACaHV and the remaining strains on BACa). The plates were incubated at 37°C, anaerobically for four days or until a confluent lawn was observed.

The simultaneous antagonism method was used to detect the ability of a producer strain (*S. salivarius*) to inhibit the growth of co-seeded indicator strains. A lawn of *P. intermedia, P. gingivalis* or *P. canoris* on either BACaHV or BACa from cells suspended in 3 ml THB to a 0.5 McFarland Standard onto a fresh agar plate. Then a pure-producer colony, which was ‘picked’ and then ‘stabbed’ into the agar plate, already inoculated with the lawn. These were incubated anaerobically, at 37°C, for 2–4 days or until the lawn of the indicator strain was confluent. The resulting zone diameter was recorded.

### Attachment of *S. Salivarius* Strains to HEp-2 Cells

*Streptococcus salivarius* strains were tested for their ability to bind to the mammalian HEp-2 cells; M18, M18^−/−^ (megaplasmid deficient), M18^K12p^ and K12^M18P^ (as derived above with exchanged megaplasmids), K12, K12^−/−^ (megaplasmid deficient), JIM8777, ATCC7073, Min5, Tove R, Tove S and JH. To assess adherence, HEp-2 cells (ATCC CCL23) were grown into a monolayer in flat-bottomed 96-well plates while *S. salivarius* strains of interest inoculated in 3 ml THB and incubated overnight, at 37°C in 5% CO_2_ in air. Overnight bacterial cultures were spun in micro-centrifuge tubes for 5 minutes. The supernatant was discarded and the cells were suspended in PBS before being centrifuged again for 5 minutes. Cells were resuspended in Dulbecco’s Modified Eagle’s Medium (DMEM) [Invitrogen, Auckland, NZ] and diluted to 5×10^5^–5×10^6^ cells/ml. To obtain an initial cell count, the samples were further diluted up to 10^−5^ and plated out using the Miles-Misra method technique (10 µl in triplicate per dilution per strain) onto 2YT agar plates [30]. The monolayers of HEp-2 cells were washed three times in PBS and then mixed with 100 µl of strain of interest into each well containing HEp-2 cells. All tests were carried out in triplicate and incubated for 3 hours at 37°C in 5% CO_2_ in air. The wells were then washed three times in PBS to remove non-adherent bacteria. To dissociate the HEp-2 cells from the wells, 30 µl of 0.5 g/l trypsin and 0.2 g/l EDTA solution was added to each well and incubated for 30 minutes at 37°C in 5% CO_2_ in air. Using an inverted microscope, cells were checked to ensure that most HEp-2 cells have been dissociated from the wells. Then 70 µl of THB was added to the wells and pipette used to dislodge the HEp-2 cells from the walls. These were then plated using the Miles-Misra technique onto 2YT agar plates and incubated at 37°C in 5% CO_2_ in air, overnight. For enumerating *S. salivarius* in cell-association assays, 2YT (yeast/tryptone) agar (2% (w/v) tryptone [Bacto, BD], 1% (w/v) yeast extract [Bacto, BD], 1% (w/v) sodium chloride, 1.5% (w/v) bacteriological agar) was used. Percentage adherence for each strain was calculated by determining the average number of CFU per dilution (as CFU/ml), this value was divided by the initial number of colonies/ml added to the corresponding well and then multiplied by 100.

### Detection of Adhesion Factors

Twenty colonies from a fresh culture on solid media were individually resuspended in 300 µl of 0.85% (w/v) NaCl in 1.5 ml microfuge tubes and were DNA extracted. The genes encoding *cspA, cspB,* orf 166 and orf 176 were investigated through PCR. The *cspA* and *cspB* products were amplified with primers (5′-3′), *cspA* forward; GCC TAA CGC TAC GGA TAC TGC TAA T, *cspA* reverse; ACT GCT CCT CCT GCC TGT GAA G, *cspB* forward; CCA ACA TAA AGG GAC ACC AAC TAC GAG, *cspB* reverse; CCC ATC CGG ATT AAC GCT ACC A. Amplification utilised an initial denaturation step at 92°C for 2 minutes, annealing at 55°C for 2 minutes, followed by elongation at 65°C for 5 minutes. This was followed by 32 cycles of denaturation at 92°C for 30 seconds, annealing at 55°C for 30 seconds and elongation at 65°C for 3 minutes. The PCR conditions for the amplification of orf 166 and orf 176 used primers Orf 166 forward; CGA GAG TTT GCT GCC ATA CA, Orf 166 reverse; GGC AAC ACC AGC GTT TTT AC, Orf 176 forward; CTT TCT CGA CAG TAA GGC GG and Orf 176 reverse; TGA AAT TCC AAC TCC TTG CC and included an initial denaturation step at 94°C for 2 minutes, followed by 30 cycles of denaturation at 94°C for 30 seconds, then annealing at 55°C for 30 seconds and elongation at 72°C for 1 minute. This was followed by a final elongation step at 72°C for 5 minutes.

### Co-aggregation Assay

To determine the ability of *S. salivarius* to co-aggregate with strains *Porphyromonas gingivalis* ATCC 33277, *Aggregatibacter actinomycetemcomitans* V29523 and *Fusobacterium nucleatum* FH2, the *S. salivarius* strains were inoculated into 10 ml THB and incubated at 37°C, in 5% CO_2_ in air, overnight. The periodontal pathogen strains were inoculated into THB and incubated at 37°C anaerobically for 2–3 days. The bacteria were harvested by centrifugation at 3000×*g* for 10 minutes and washed three times in 1 ml volumes of aggregation buffer (0.121 g Tris, 0.022 g calcium chloride, 0.031 g hydrated magnesium chloride, 8.766 g sodium chloride/L) to ensure complete removal of culture medium. The cell suspensions were diluted 100-fold with aggregation buffer and 500 µl aliquots of each periodontal pathogen was individually mixed with 500 µl of each of the test *S. salivarius* strains. The turbidity of the mixtures were recorded at 15 minutes, 40 minutes and 8 hours, and given a score depending on the aggregation.

## Results

### Indigenous *S. salivarius* Strains Commonly Produce Bacteriocins

**Figure 1:**
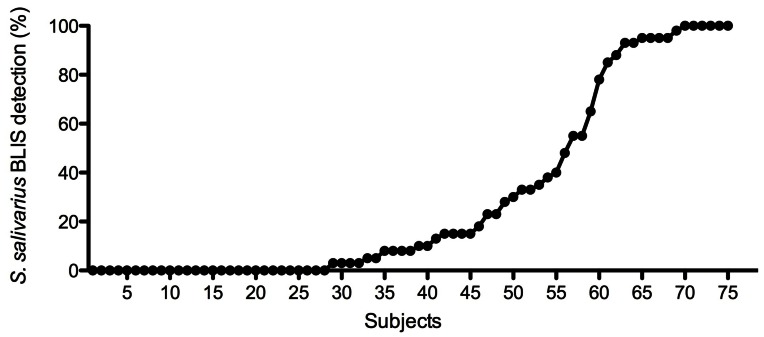
Total proportion of bacteriocin-like inhibitory substance producing *Streptococcus salivarius* in saliva samples in all subjects tested prior to probiotic treatment.

Sixty two per cent of subjects had BLIS-producing *S. salivarius* detected in their saliva prior to dosing and in 21% of these individuals the BLIS producers represented >80% of the total *S. salivarius* population (Figure 1). Further analysis of this BLIS activity using the P-typing method [20] showed that 26% of subjects had *Streptococcus salivarius* which produced antimicrobial substances, which inhibited at least 3/9 indicator organisms, tested (data not shown). None of the subjects carried *S. salivarius* strains having inhibitory activity against *Streptococcus mutans* OMZ 175, an organism sensitive to the bacteriocin produced by strain M18, as tested against 40 isolated *S. salivarius* colonies from each subject (data not shown).

### *Streptococcus Salivarius* M18 Oral Persistence Appears Partially Dose Dependent

**Figure 2:**
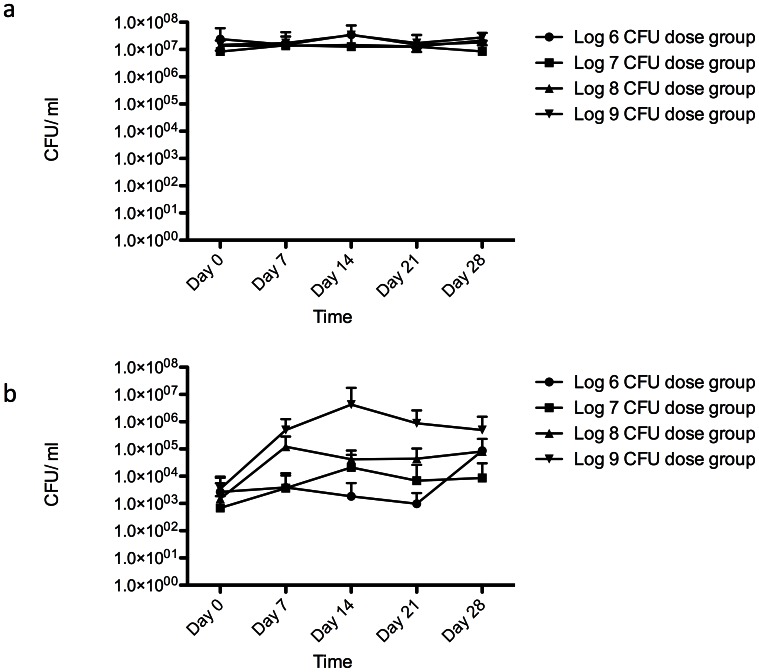
a. *Streptococcus salivarius* colony forming units obtained on Mitis Salivarius agar over duration of the study from the saliva of subjects that received differing doses of *S. salivarius* M18 (error bars denote ±SD). b. *Streptococcus salivarius* colony forming units obtained on Mitis Salivarius agar containing streptomycin as also used as a selective marker for the probiotic strain (error bars denote ±SD).

No differences in the total mean salivary *S. salivarius* (CFU/ml) for each subject was found from baseline during the course of dosing with strain M18 (Figure 2a). To enumerate strain M18, saliva was plated on CABCa agar supplemented with 100 µg/ml streptomycin. An average background level of approximately 1×10^3^ CFU/ml of naturally resistant *S. salivarius* was detected in the baseline samples (Figure 2b). However, after dosing with M18, the levels of streptomycin resistant *S. salivarius* increased substantially, indicative of the strain’s presence within the salivary microbiota. The exception was the lowest dosage group, which only showed an increase in streptomycin resistant *S. salivarius* in the 4-week sample. Progressive increases in strain M18 dosage resulted in correspondingly greater proportions of streptomycin resistant *S. salivarius* in the salivary population. In the highest dosage group (1×10^9^ CFU/dose/day), the putative M18 colonisation levels were one log higher than in the other dosage groups.

**Figure 3:**
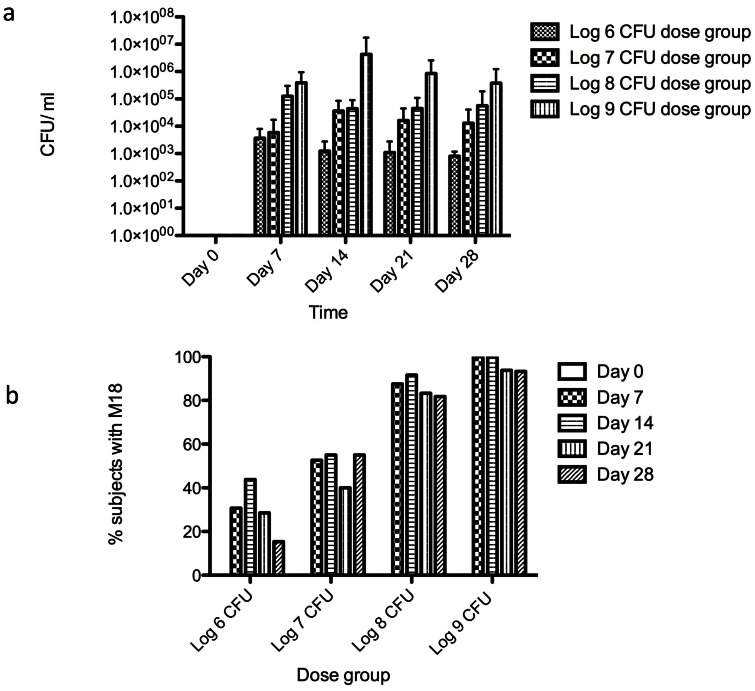
a. Mean number of *S. salivarius* M18 detected in saliva samples at different time points receiving different probiotic doses (error bars denote ±SD). b. Percentage of subjects with *S. salivarius* M18 detected in saliva samples.

The log counts showed that subjects who received higher probiotic dose retained higher numbers of M18 (Figure 3a). The percentage of subjects having the M18 strain detected in their saliva increased with the dose quantity (Figure 3b). However, after day seven, the salivary probiotic numbers did not appear to increase, despite further dosing. The trend was for the cell numbers to slowly track downwards.

### V6 Region 16S rRNA Sequence Analysis of the Salivary Microbiota does not Indicate Major Perturbations by Probiotic Instillation

**Figure 4:**
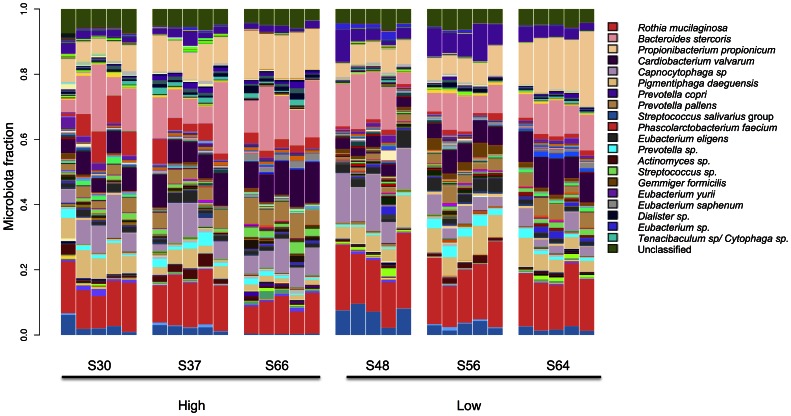
Each bar represents a single saliva sample and each cluster of bars is a single participant (starting at time 0 and sampled once a week for up to 4 weeks total). The colored segments represent the relative fraction of each bacterial taxon detected at 1% relative abundance or greater (twenty most predominant OTU shown). Sequences at less than 1% abundance have been included in the ‘‘remainder’’ fraction at the top of the bar (see color legend of bacterial taxa).

**Figure 5:**
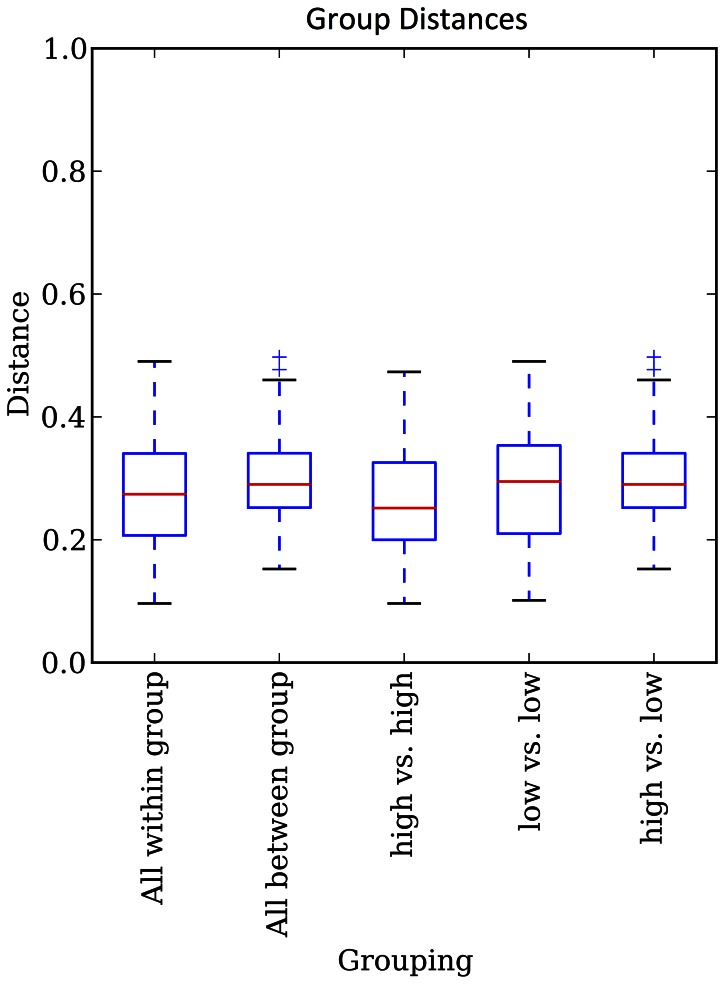
Diversity of the salivary microbiota and if either high or low M18 probiotic persistence had a significant impact. Box plots show weighted UniFrac distances.

To determine whether instillation of an exogenous organism influenced the composition of the salivary microbiota when persisting at different levels, six subjects that exhibited the most consistently low or high levels of M18 salivary levels had their microbial composition examined in greater detail. The average strain M18 salivary persistence levels were log 5.34 for the subjects in the high persistence group compared to 4.15 for the those in the low persistence group (P = 0.0026). 16S rRNA gene V6 region amplification and sequencing yielded an average of 258, 210 sequences per sample. These were consigned to 197 operational taxonomic units (OTU) based upon grouping sequences using a 95% DNA sequence identity cutoff. The number of sequence reads per OTU was converted to the total proportion per sample. The first five OTU groups accounted for more than 50% of the total sequences and the top ten accounted for over 70% of sequence types. Analysis of the composition of all samples from the subjects indicated no significant ecological shifts in the microbiota following the probiotic dosing by weighted UniFrac analysis (Figure 4 & 5). The relationships between the samples predominantly appeared to cluster by participant; that is, a subject’s microbiota was most similar to itself at all time points than it was compared to any other subjects whether in either the ‘high’ or ‘low’ groups (Figure 5).

### Megaplasmid Transfer Results in a Recipient Strain with Hybrid Characteristics

All the *S. salivarius* strains tested, except strain NR, had inhibitory activity against *P. intermedia*. The plasmids of strains K12 and M18 must harbour the BLIS activities against strains ATCC25611 and BGBL, as their plasmid-negative derivatives lost the ability to inhibit these same strains (Table 1). Inhibition of *P.* *gingivalis* strains JK45, W50, and *P. canoris* strain P21 was media-dependent with inhibition on either BaCa or BaCaHV agar, depending on the indicator and producer strain (Table 1). After prolonged incubation of the indicators (up to seven days), the zones of inhibition were no longer visible, indicating that the initially observed inhibitory activity may have been bacteriostatic. Some synergy between chromosomal and extra chromosomally encoded elements exist as after megaplasmid transfer some of the antimicrobial activities were different in the transconjugants, for example M18 and K12 wild type strains both inhibited strain JK45 on BACa, but neither of the transconjugants displayed this activity on the same media.

**Table 1:** Simultaneous antagonism of *S. salivarius* wild type and modified strains against selected oral bacteria.

		Simultaneous inhibition of indicator strain^a^ and agar type tested							
		*P. gingivalis* JK45		*P. gingivalis* W50		*P. canoris* P21		*P. intermedia*ATCC 25611	*P. intermedia* BGBL
Producer strain	P-Type	BACa	BACaHV	BACa	BACaHV	BACa	BACaHV	BACa	BACa
K12	777	+++	+++	+	+	+++	+++	++	+
K12^−/−^	000	−	+++	+	+	+	++	−	−
M18	677	+++	+++	++	+	++	+++	+++	++
M18^−/−^	000	−	−	+	−	−	++	−	−
M18^K12p^	777	−	++	++	+	+++	+++	++	+
K12^M18p^	677	−	–	+	+	+	++	++	−

a diameter of inhibition zones (mm) – “−”, no inhibition; “+”, ≤3 mm; “++”, ≤5 mm, “+++”, ≤7 mm.

### Multiple Factors are Involved in the Attachment of M18 to Host and Bacterial Cells

**Figure 6:**
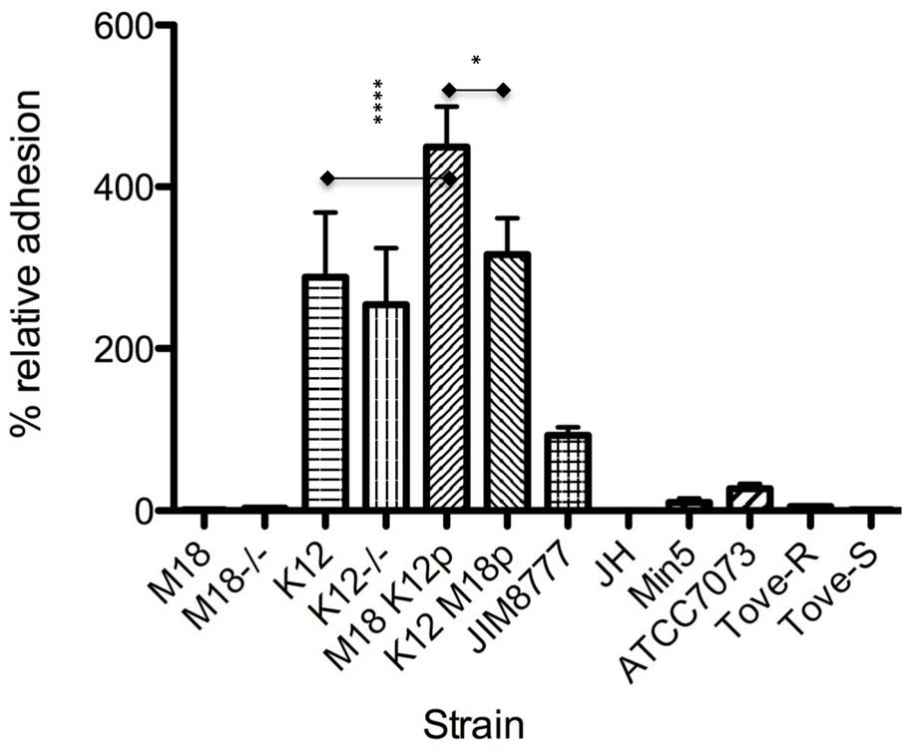
Relative adhesive abilities of *Streptococcus salivarius* strains to HEp-2 cells with different megaplasmid combinations. (Error bars denote ±SD, the method of Turkey was used for multiple comparisons (*p<0.05, **p<0.01, ***p<0.001) and significance shown where the K12 megaplasmid was inserted into non adherent M18 strain.

Neither M18 nor its megaplamid-cured derivative could adhere to HEp-2 cells. However, strain K12 (and its variant strains including the megaplasmid deficient K12^−/−^ and the transconjugant containing the M18 plasmid K12^M18p^), adhered well *in vitro* (Figure 6). Strain M18^K12p^ had a relative adherence in excess of 400%, whereas, both the wild type and cured derivatives of strain M18 had adherence scores of fewer than 10%. Previous studies have shown that it is not possible for the HEp-2 cells to internalise M18 (Burton unpublished) and therefore all values were considered adherence. Previous studies have looked at *S. salivarius* adhesion to other bacteria and the host epithelium at the cellular and molecular level [31], [32]. To elucidate why the change of adhesion characteristics of the wild type strains and subsequent change upon megaplasmid loss or acquisition, strains of *S. salivarius* were evaluated by PCR for their presence or absence of genes associated with these properties, namely *cspA*, *cspB*, *orf* 166 and *orf* 176 (Table 2). Strains positive for *cspA* also have *cspB*, but did not necessarily possess *orf* 166 and *orf* 176. Strangely, *cspA* and *cspB* were not detected for the K12^M18p^ strain which may indicate modification or deletion post transfer of the megaplasmid in this case.

**Table 2:** The distribution of *cspA*, *cspB*, *orf* 166 and *orf* 176 in *S. salivarius* strains having differing adhesion capabilities to HEp-2 cells.

Strains	*cspA*	*cspB*	*orf* 166	*orf* 176	Relative adherence to HEp-2 cells (%)
M18^K12p^	+	+	+	+	449
K12^M18p^	−	−	−	−	316
**K12**	**+**	**+**	**+**	**+**	**288**
K12^−/−^	−	−	−	−	254
JIM8777	−	−	−	−	93
ATCC7073	−	−	−	−	27
Min5	+	+	−	+	10
Tove-R	−	−	−	−	5
M18^−/−^	−	−	−	−	3
**M18**	**+**	**+**	−	−	**1**
Tove-S	+	+	−	+	1
JH	+	+	−	+	0

“+” indicates positive for the gene and “−” indicates negative for the gene. Bolded strains are wild type.

All *S. salivarius* strains co-aggregated to some extent with *P. gingivalis* ATCC 33277 (Table 3). Strain M18, containing the pSsalK12 plasmid had higher co-aggregation capability than either the parent or plasmid-free variant. None of the *S. salivarius* co-aggregated with *A. aggregatibacter* V29523, and only ATCC 7073 (a fibrillated *S. salivarius* strain used as a positive control) co-aggregated with *F. nucleatum* FH2.

**Table 3:** The extent of co-aggregation of *S. salivarius* to periodontal organisms.

S. salivarius strain	Co-aggregation of periodontal pathogens as a function of time^a^									
	Fusobacterium nucleatum FH2			Aggregatibacter actinomycetemcomitans V29523			Porphyromonas gingivalis ATCC 33277			
	15 min	40 min	8 hr	15 min	40 min	8 h	15 min	40 min	8 h	
ATCC 7073^T^	(++)	(++)	+++	−	−	−	(++)	(++)	+++	
M18	−	−	−	−	−	−	+	(+)	(+)	
M18^K12p^	−	−	−	−	−	−	(++)	(++)	++	
M18^−/−^	−	−	−	−	−	−	(−)	(−)	+	
K12	−	−	−	−	−	−	++	++	++	
K12^M18p^	−	−	−	−	−	−	++	++	++	
K12^−/−^	−	−	−	−	−	−	+	++	++	

“–“ Indicates no co-aggregation, evenly turbid suspension, “+’”weak precipitation with evenly turbid supernatant, “++”moderate precipitation with evenly turbid supernatant and evidence of flocculation “+++”substantial precipitation with clear supernatant and some flocculation, () brackets indicates half-way between number of indicated ‘+’ and the number above it.

## Discussion

In the present study, BLIS-producing *S. salivarius* were shown to be a very common component of the indigenous salivary microbiota and indeed the majority of individuals yielded isolates displaying some inhibitory BLIS activity. Previous studies on pharyngitis, tonsillitis, dental caries and cystic fibrosis have shown a correlation between a reduction in the levels of potential bacterial pathogens and the presence of these “antagonistic” streptococcal commensals within the upper respiratory tract microbiota [33], [34], [35], [36], [37], [38], [39], [40]. However, probiotic application of BLIS-producing bacteria for prevention of infection has been limited [14]. There is some evidence that these probiotic organisms can confer the same level of protection as naturally harboured strains [40], [41], [42]. While antimicrobial activities may not be the only mechanism involved in host protection by indigenous *S. salivarius*, achieving persistence of probiotics likely requires mimicking the natural conditions, which is likely to be important in conferring protection.

Persistent low-level dosing did not appear to lead to cumulative increases in the proportion of the probiotic within the indigenous salivary population. However, the levels of persistence increased with higher doses. This has implications for the clinical applications of probiotics, which are often recommended to be given daily, because most do not colonize the host. Plus, their concentrations are invariably not high (apart from VSL#3 for inflammatory bowel disease), so they are often administered over the course of a day. The ability of a probiotic to persist at the target site likely allows the organism to have a greater impact on the host.

The total number of *S. salivarius* detected in the saliva of an individual did not appear to be increased above the baseline level, even when doses of up of 100 times the background levels of indigenous *S. salivarius* were administered. This implies that there is a limited capacity for the host to increase its microbiota species load. It also raises the question of what mechanisms pathogens use, and that are not present in probiotic or commensal strains, to integrate or by-pass the indigenous microbiota.

In some subjects there clearly was a high level of persistence of the bacteriocin-producing probiotic strain. Indeed, in some subjects a substantial proportion of the original *S. salivarius* population was replaced. 16S rRNA gene sequence analysis of subject’s samples with either high or low probiotic persistence did not show remarkable change in the predominant microbial composition. This echoes the findings of McNulty *et al.* (2011) who showed that probiotic yogurt did not alter the gut microbiota [43]. The microbiota of the young adults tested here seemed to be relatively robust with regards to perturbation by probiotic instillation (Figure 4). While there were some bacterial types that were significantly different in their proportions both compared to pre samples and between the two persistence groups, these changes did not represent major perturbations of the microbiota. The absence of such disturbances actually supports the safety of strain M18, as regulatory agencies view disruption of the indigenous microbiota of healthy subjects as undesirable.

Our group [8] has demonstrated the transfer of bacteriocin-encoding plasmids into indigenous oral *S. salivarius* strains. The present study has demonstrated the first *in vitro* transfer of bacteriocin-encoding megaplasmids between two strains of *S. salivarius*. This opens up the possibility of creating tailor-made probiotic strains through the transmission of megaplasmids from poorly-persisting, antimicrobial producing *S. salivarius* strains into poor bacteriocin-producing but strongly persisting indigenous *S. salivarius*, strains potentially conferring better protection to the host.

Adhesion is an essential first step for any colonisation of the oral cavity since non-adherent bacteria will be rapidly washed away in the salivary flow. The binding of bacteria to tissue cells involves specific adhesins [44], [45]. These include proteins that contain a LPXTG motif (membrane anchor) near the C terminus [46], such as *cspA* and *cspB* (cell-surface protein A & B) [32]. Lévesque *et al.* [32] suggested that *cspB* may be associated with fimbriae, and thus could contribute to adhesion specificity, as fimbriae have been noted to be key components of the cell-to-surface and cell-to-cell adhesion of oral bacteria [47]. Another surface component, the fibrils are also important for adhesion of streptococci to oral surfaces and co-aggregation with other oral bacteria [31], [48].

In the present study, CspA and CspB proteins were found to be plasmid-encoded on M18 and K12, but was also detected the poorly adhering Tove-S, ATCC25975 and JH strains. Levesque *et al.* [32] suggested that the Orf176 protein might also be a transcriptional regulator for *cspA* and *cspB* and also *orf166*, which encodes cell-surface protein [49]. Only strains M18^K12p^ (with the pSsalK12 megaplasmid) and wild-type K12 possessed all four genes. *Orf166* may have a role in the adhesion of *S. salivarius* to HEp-2 cells since strain M18^K12p^ had a high relative adherence to HEp-2 cells but strain M18 showed poor adhesion. This suggests that *Orf166* helps to mediate the adhesion process and in future studies deficient derivatives may assist in determining this. However, as strain K12^−/−^ also adhered strongly to HEp-2 cells, so other factors must also promote K12 adhesion.

Co-aggregation has been documented for strains of the oral cavity [50], [51], including *S. salivarius* and periodontal pathogens [52]. It has been suggested that *S. salivarius* co-aggregation with potential pathogens may be a means of eradicating the reservoir of the salivary anaerobes [53]. Of the *S. salivarius* strains tested here, only ATCC 7073 (a fimbriated K^+^ strain, carrying fibrils) was able to co-aggregate with *F. nucleatum*. This is interesting as it implies a degree of specificity between strains not due to fimbriae, as the fimbriated K^-^ strain, ATCC 25975 was unable to bind to *F. nucleatum* This finding was similar to that of Levesque *et al.* [52] except in their case, a *F. nucleatum* strain co-aggregated with strain ATCC 25975, as well as a fimbriae-negative mutant of strain ATCC 25975 called strain D37. This further rules out a role for fimbriae in coaggregation.

In conclusion, this study has shown that the persistence of a probiotic *S. salivarius* strain in the mouth was dose dependent. This ability to persist could allow the probiotic the opportunity to more effectively counter pathogens as well as inducing host gene expression pathways of homeostasis and cytoskeletal repair in the epithelial lining [54], [55]. The use of high dose M18 to treat and prevent oral diseases, in comparison to antibiotics, warrants further clinical testing with the hope of providing alternative option in dental practice.
